# Seasonal Variations in the Gut Fungal Communities of Hooded Crane (*Grus monacha*) at Wintering and Stopover Sites in China

**DOI:** 10.3390/ani11040941

**Published:** 2021-03-26

**Authors:** Nazia Mahtab, Lizhi Zhou, Fengling Zhang, Wei Wang

**Affiliations:** 1 School of Resources and Environmental Engineering, Anhui University, Hefei 230601, China; naziaecology@gmail.com (N.M.); 17856516528@163.com (F.Z.); wangwei.edu.0551@gmail.com (W.W.); 2 Anhui Province Key Laboratory of Wetland Ecosystem Protection and Restoration, Anhui University, Hefei 230601, China

**Keywords:** hooded crane, migration, seasonality, fungi, high-throughput sequencing, pathogens

## Abstract

**Simple Summary:**

The gut fungi assist the host in various physiological activities, homeostasis, immune responses, and growth. The diversity and community composition of gut fungi are driven by multiple factors, including diet, environmental exposure, habitat type, and seasonal migration. Migratory birds have a peculiar life cycle, so it is interesting to understand the ecological function of their “gut fungal microbiome.” Birds are exposed to variable diets, environments, and habitats amid seasonal migration. The hooded crane is known as a long-distance migratory bird, inhabiting both wintering and stopover grounds during seasonal migration. During migratory seasons, it inhabits various habitats and is exposed to variable environments. This study analyzed the shifts between gut fungal diversity and the community composition of the hooded crane at both wintering and stopover sites amid seasonal migration. The gut fungal alpha diversity exhibited a more significant change during winter than in fall and spring. The gut fungal community composition exhibited significant shifts across winter, fall, and spring (ANOSIM, *p* = 0.001). The pathogenic diversity and relative abundance showed significant differences during winter at the wintering site relative to fall and spring at the stopover site. Moreover, the pathogenic fungal community composition was significantly different during fall, winter, and spring. This work contributes to present essential knowledge about the gut fungal microbiome of hooded cranes amid seasonal migration. This study also implicated that conservation measures for hooded crane conservation should be applied, as the risk of cross-transmission of potential fungal pathogens might increase during seasonal migration.

**Abstract:**

The “gut fungal microbiome” maintains the immune system, homeostasis, and various physiological functions of an organism. Different factors shape and affect gut fungal diversity and community composition, such as environment, habitat type, food resources, and seasons during migration. Wild birds amid migration are exposed to different habitats with different environments, available food resources, and seasons, which may substantially impact their gut fungal community composition and diversity. The hooded crane (*Grus monacha*) is a known migratory bird that migrates over long distances and is exposed to varied habitats with different environments and food types. We investigated the differences in gut fungal diversity and community composition between wintering and stopover sites amid three migratory seasons. We deduced the gut fungal pathogenic diversity and community composition during winter, fall, and spring by using high throughput sequencing (Illumina Mi-seq), and the internal transcribed region 2 (ITS2) was examined. Samples were collected from Shengjin Lake in the winter and Lindian during the fall and spring. The dominant fungal phyla found across the three seasons were Ascomycota, Basidiomycota, Zygomycota, and Rozellomycota. The gut fungal alpha diversity showed significant shifts during winter at the wintering site compared with the fall and spring seasons at the stopover site. The fungal community composition exhibited a significant change across the three seasons (ANOSIM *p* = 0.001). The results also demonstrated that the diversity and relative abundance of potential pathogens also showed divergence in winter compared to fall and spring. This study provides the basis for understanding the discrepancy in gut fungal diversity and community composition during migratory seasons at both wintering and stopover grounds. It also suggests that conservation measures should be applied to the conservation of hooded cranes and other wild birds, as the risk of cross-infection increases during seasonal migration.

## 1. Introduction

The gut provides a suitable environment for a diversity of microbes, more importantly, bacteria with a small proportion of fungi, protozoa, virus, and archaea [1,2]. Gut microbial studies have been conducted for many animal species, and gut bacterial communities cover a broad range of studies. Little information is present about the “fungal microbiome” (fungi) of wild birds. It has been elucidated that various commensal, symbiotic, and opportunistic pathogenic fungi reside in the host gut [3]. The gut fungal communities modulate gut homeostasis, interact with other microbes in the gut, and produce some essential metabolites. These metabolites contribute to immune development and balance the gut physiological functions [4]. Some fungal genera in the gut contribute to digestion and regulate the host’s digestion efficiency [5]. Gut fungi provide crucial amino acids and facilitate lignocellulose breakdown in the host gut [6]. The disruption in gut ecology causes an imbalance in gut microbial communities, allowing the fungal pathobionts to produce pathological effects. Some potential fungal pathogens are carried through host-environment interactions [7]. Moreover, many factors influence gut fungal diversity and community composition, such as host genetics, age, sex, lifestyle, diet, seasonal changes, immune system, and disease condition [8].

The life cycle of migratory birds is diverse, pertaining to seasonal migration with exposure to different environments, food resources, and habitat types. It is interesting to study the gut fungi of wild birds during seasonal migration. Gut fungi promote the absorption of nutrients from various food resources and trigger their host’s immune function [9]. Birds during migration require more energy for the continuation of their migratory flights. The difference in birds’ food resources at wintering and stopover sites during seasonal migration allows the gut microbiota to shift to fulfill the high-energy demands of migration [10]. During different seasons at wintering and stopover sites, wild birds forage on food resources exhibiting different nutrient contents. Therefore, the gut fungi of wild birds might help them to acquire energy by the absorption of nutrients from various food resources available at wintering and stopover sites during seasonal migration [6]. Moreover, it is interesting to study the hooded crane’s gut fungal microbiome to demonstrate its ecological function of enhancing digestion efficiency during seasonal migration. Isolation methods and traditional culturing methods are time intensive and require specific media for fungal growth. Thus, the developed molecular technique of the next-generation sequencing approach helped in our gut fungal detection and analysis [11].

The hooded crane (*Grus monacha*) is one of the long-distance migratory birds. It is determined as a vulnerable (VU) species in the Red List of Threatened Species [12], and in China, it is defined as Category I key National Protected Wild Animal Species. The hooded crane has three-fixed stopover sites during fall and spring migration in several areas including South Korea, Russia, and the Songnen Plain regions in Northeast China [10]. Mainly, breeding grounds for the cranes are present in South Siberia. The wintering grounds are situated in Japan and South Korea and middle and lower Yangtze River floodplain in China [13]. The wintering population in China approximately consists of 1050–1150 individuals [12]. Empirical studies indicated that the cranes spend a long winter period (November to April) at wintering grounds and a comparatively short time period (14 days) during each fall and spring migration at the stopover sites [10].

Shengjin Lake is located in the middle and lower Yangtze River floodplain, which is considered as one of the crucial wintering areas for hooded cranes [14]. It is also an important wintering ground for a large number of migratory birds during the wintering period. The hooded crane mainly feeds on some dominant plants, i.e., *Potamogeton crispus*, *Ceratophyllum demersum*, *Carex thunbergii*, *Potentilla supina*, *Polygonum criopolitanum*, *Ranunculuc polii*, rice grains, and invertebrates such as mollusks [15]. During winter, Shengjin Lake faces degradation due to human interference, which alters the food availability for cranes. Because of the low availability of foraging resources, the hooded crane alters its dietary structure to balance its energy levels during the harsh winter period. The hooded crane shares the same feeding niche with domestic poultry and other wild birds at wintering sites. The sharing of the same foraging niche might increase the cross-transmission of gut microbiota and potential pathogens at wintering sites [16]. Recent studies demonstrated that hooded cranes encounter severe living conditions in the winter with food shortages, which encourages changing their foraging habitats and dietary structure. During winter, hooded cranes rely more on their gut fungal microbiome to increase the gut digestion function and their immunity against potential fungal pathogens [6]. Lindian is one of the important stopover sites for hooded cranes, located in Songnen Plain, Heilongjiang Province, occupying areas suitable for cranes with regard to food resources. It is a stopover site that 34% of the population occupies during fall and spring migration [17]. Mainly, the available food resource is corn because the annual crop grown at this site is corn, which ripens in fall. Therefore, the availability of corn might be higher in fall than in spring in the same habitat. Hooded cranes might also be exposed to variable environment and food availability before arriving at stopover sites during fall and spring migration [10]. Consequently, at the same site during different seasons, the gut fungal community composition may shift in hooded cranes.

Exposure to a diverse environment, food availability, abundance, and habitats may cause significant shifts in the gut fungal microbiome during fall, winter, and spring. We conducted this study to identify the core “gut fungal microbiome” of hooded cranes across winter, fall, and spring, and to evaluate our hypotheses: (a) availability of various food resources at different habitats may cause a significant difference in the diversity and community composition of the “fungal microbiome” at wintering (during winter) and stopover sites (during fall and spring); (b) seasonal differences may cause a difference in food abundance at the same habitat for the hooded crane; therefore, the gut fungal community composition may differ between the fall and spring stopover sites; (c) the shared foraging environment of the hooded crane during the extended wintering period with domesticated poultry and other wild birds may increase the cross-transmission of potential fungal pathogens at wintering sites.

## 2. Materials and Methods

### 2.1. Study Site and Sample Collection

**Figure 1:**
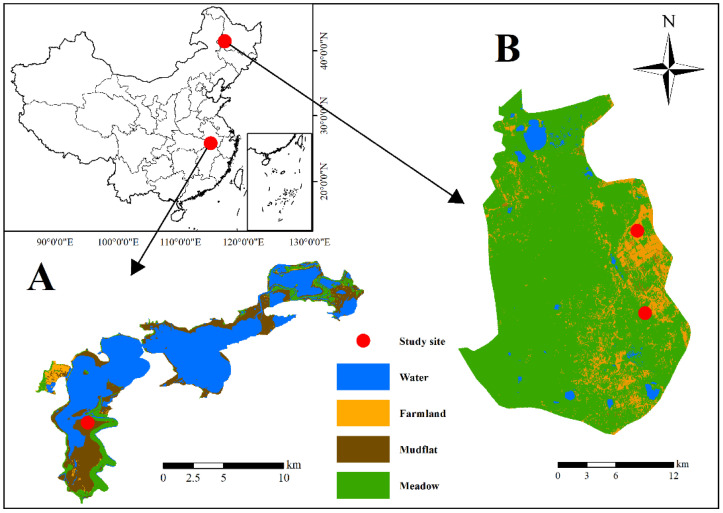
Area of fecal sample collection of the hooded crane from (**A**) Shengjin Lake and (**B**) Lindian.

Samples were collected from Lindian (46.73~47.48° N, 124.3~125.35° E), which is an important stopover site of the hooded crane, situated near the Wuyu’er River on the West Songnen Plain [17] and Shengjin Lake (30.25~30.50° N, 116.92~117.25° E), an important wintering ground present in the middle and lower of Yangtze River floodplain [18,19] (Figure 1).

This study included a total of 60 samples over three periods. A total of 20 fecal samples for each period, fall (October 2017) and spring (March 2018), were gathered from Lindian, respectively. At Shengjin Lake, a total of 20 fecal samples were collected during winter (January 2018). A non-invasive sampling method was applied following the sample standards outlined in previous studies [10]. Before the sample collection, the birds were first observed and then sampled after they completed their foraging. The areas with more than 50 birds were selected as sampling areas, and each sample was collected at intervals of 5 m. Gloves were used to avoid sample contamination, and samples were stored in sterile bags and preserved on ice before using them for lab analysis [6].

### 2.2. DNA Extraction from Fecal Samples

Using and following the extraction protocol given on Qiagen DNA Stool Mini Kit, fecal DNA was extracted. The DNA load in each extracted sample was assessed by using NanoDrop ND-1000 (Thermo Fisher Scientific, Wilmington, DE, USA). Extracted DNA was preserved at –80 °C to be used for further analysis.

### 2.3. Species Identification

The bird mitochondrial gene cytochrome C oxidase subunit 1 (COI) was amplified by using the BIRDF1(5′-TTCTCCAACCACAAAGACATTGGCAC-3′) and BIRDR1 (5′-ACGTGGGAGATAATTCCAAATCCTG-3′) primer sets to determine the hooded crane species [6]. A PCR test was conducted by following the listed parameters: 95 °C for 5 min, followed by 35 cycles of 95 °C for 30 s, 55 °C for 45 s, and 72 °C for 90 s, with a final extension period at 72 °C for 10 min. The end products of the PCR were applied for Sanger sequencing and were identified by Basic Local Alignment tool (BLAST) in the National Center for Biotechnology Information (NCBI) database with (>97% sequence identity) [10]. Identified samples that belonged to the hooded crane species were further used for high-throughput sequencing.

### 2.4. Library Preparation

The extracted DNA was transported after species identification to Majorbio (Shanghai, China) for next-generation sequencing (Illumina Mi-seq) of the internal transcribed spacer 2 (ITS2) gene. The ITS2 gene is a subregion of nuclear ribosomal and is present as a DNA spacer between 5.8S and 28S small subunit rRNA. It is known as a universal novel DNA barcode to identify fungal taxa in a community. It possesses valuable characteristics, i.e., conserved regions to design primers, short-sequence length, ease in amplification, and aids to distinguish closely related species [20]. PCR was performed using primer set ITS3 forward primer (5′-GCATCGATGAAGAACGCAGC-3′) and ITS4 reverse primer (5′-TCCTCCGCTTATTGATATGC-3′) to amplify the ITS2 gene for gut fungal analysis. [21].

### 2.5. Data Analysis

Raw fungal data were obtained by Quantitative Insights into Microbial Ecology (QIIME V.1.9) (Knight and Caporaso labs, Colorado, USA) [22]. Raw reads were cleaned to attain good-quality effective sequences by applying the deblur algorithm [23]. For clustering unstructured data UCLUST algorithm with (~97% similarity,) was used to cluster good-quality sequences into operational taxonomic units (OTUs) [24]. Within each OTU, the most abundant sequences were chosen as representative sequences, and sequences were classified taxonomically by using UNITE a web-based database and sequence management environment for molecular identification of fungi by targeting fungal ITS2 gene. Python Nearest Alignment Space Termination (PyNAST), sequence aligner, was used to align the obtained sequences [22]. For equal rarefication within samples, subsets of 25,000 reads were collected randomly, with repetition of ≥20 for each sample for comparing fungi and community diversity. To analyze the species diversity and richness, rarefied samples were used by evaluating alpha diversity indices (OTU richness and Chao1 diversity index). Data in raw format was submitted into NCBI Sequence Read Archive (BioProject ID: PRJNA697866).

### 2.6. Pathogenic Species Determination

All the fungal species determined were probed in the Web of Science database and Google Scholar to determine the pathogenic fungi. Species ascribed as pathogenic in humans, other animals, and plants were subjected to further analysis and considered in the related literature (Table S6).

### 2.7. Statistical Analysis

One-way analysis of variance (ANOVA) and the Kruskal–Wallis test for normally distributed data (*p* > 0.05) and non-normally distributed data with (*p* < 0.05), respectively, were applied to analyze the alpha diversity and relative abundance of the dominant fungi (>1%). Mainly, the Kolmogorov–Smirnov test was used to evaluate the normality of data (Table S1). The hooded crane gut fungal biomarkers in each season were estimated by linear discriminant analysis (LDA) effect size (LEfSe) (Figure S1) [25]. LEfSe analysis was performed through galaxy flow work (Galaxy, The Huttenhower Lab; https://huttenhower.sph.harvard.edu/galaxy/) (accessed on 11 March 2021) to determine the significant difference between the gut fungal taxa during winter, spring, and fall. In R software (V.3.1.0) (R Core Team, Vienna, Austria), the “vegan” package was applied to compare groups across seasons using one-way similarity with permutations of 999. The difference in the fungal and pathogenic community composition between the three seasons was estimated by non-metric multidimensional scaling (NMDS) and analysis of similarity (ANOSIM; permutation = 999) using the “vegan” package in R software [26]. The “labdsv” package in R software (V.3.1.0) was applied to determine the indicator OTUs. Similarity Percentage (SIMPER) analysis was used to assess the contribution of specific OTUs to dissimilarity across each sampling season [27]. The pathogenic species’ relative abundance during the three seasons was estimated by applying the Kruskal–Wallis test followed by non-normal distribution.

## 3. Results

### 3.1. Gut Fungal Alpha Diversity

**Figure 2:**
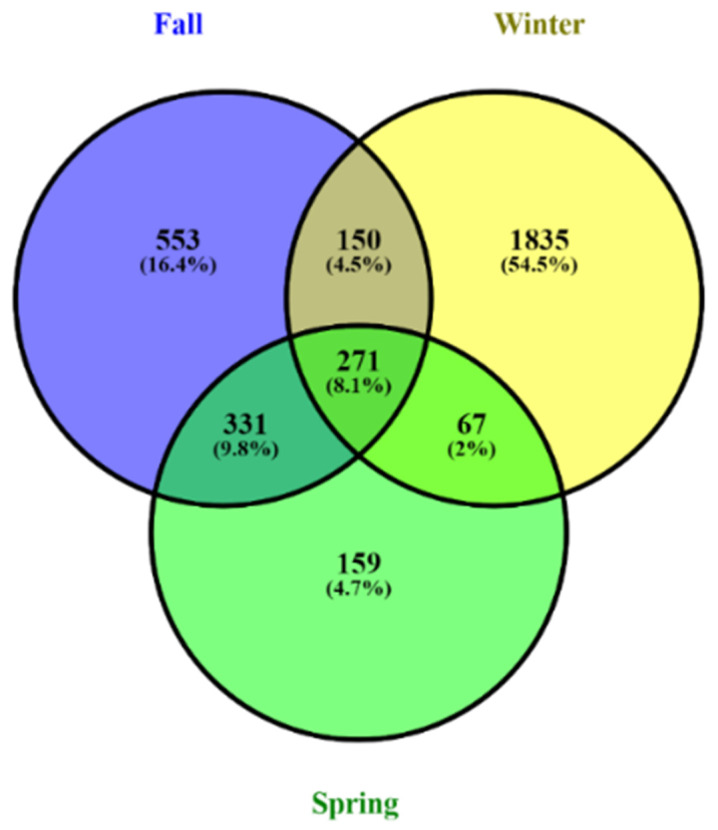
Analysis indicates the unique and common gut fungal operational taxonomic units (OTUs) of the hooded crane during fall, winter, and spring using a Venn diagram.

A total of 3,623,384 high-quality fungal sequences were obtained over all samples, ranging from 29,236 to 74,354 per sample (Table S7). A total of 3366 OTUs were obtained in the range from 57 to 598 (97 % similarity); 271 (8.1%) were present in all seasons. The number of shared fungal OTUs during fall and winter was 150 (4.5%). The number of shared OTUs between winter and spring was 67 (2%). During fall and spring, the shared OTUs number was 331 (9.8%). The unique OTUs during fall, winter, and spring were 553 (16.4%), 1835 (54.5%), and 159 (4.7%), respectively (Table S2, Figure 2).

**Figure 3:**
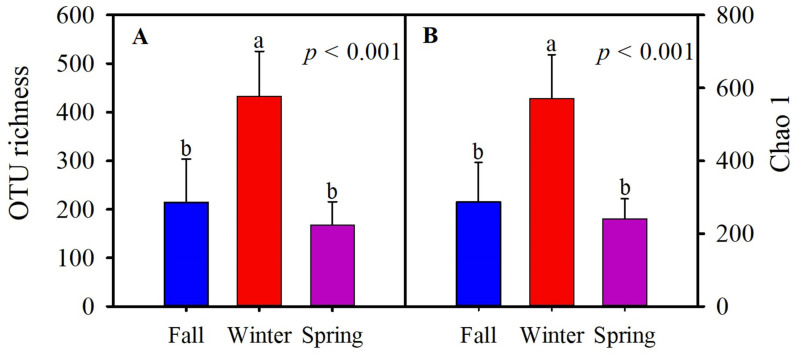
Gut fungal alpha diversity of the hooded crane during three seasons. (**A**) Operational taxonomic unit (OTU) richness, and (**B**) Chao 1. Bars exhibit mean value; error bars exhibit standard deviation; the different letters ‘’a’’ and ‘’b’’ over the bars represent significant differences from one-way ANOVA by applying Tukey’s Honest Significant Difference HSD test (*p* < 0.05). OTU, operational taxonomic unit.

The fungal alpha diversity was evaluated by the OTU richness and Chao1 data of the hooded crane for three seasons. Relative to fall and spring, the alpha diversity was higher in winter (Figure 3).

### 3.2. Gut Fungal Community Composition

**Figure 4:**
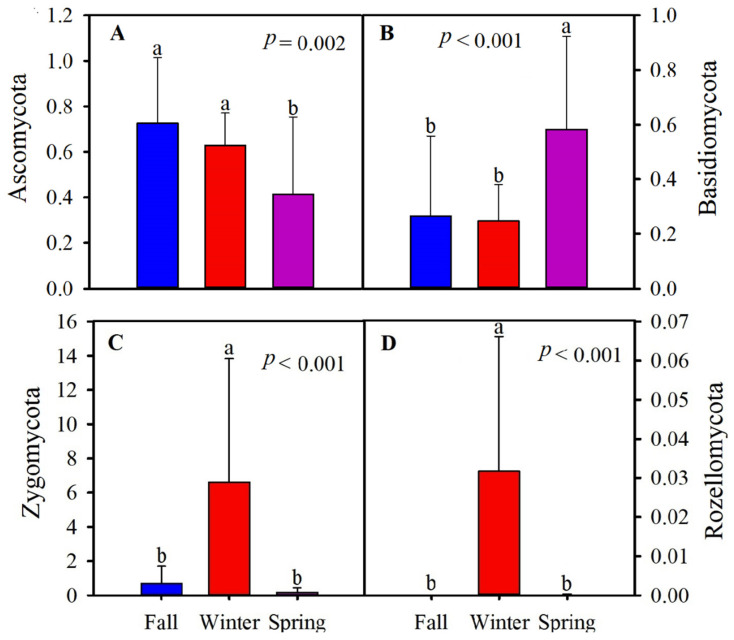
The relative abundance of the dominant gut fungal phyla of the hooded crane in fall, winter, and spring. (**A**) Ascomycota, (**B**) Basidiomycota, (**C**) Zygomycota, and (**D**) Rozellomycota. The different letters ‘’a’’, ‘’b’’ over bars represent significant differences in Tukey’s HSD comparisons (*p* < 0.05). Bars represent mean; error bars indicate standard deviation.

Ascomycota (58.98%), Basidiomycota (36.49%), Zygomycota (2.50%), and Rozellomycota (1.06%) were evaluated as dominant gut fungal phyla during the three seasons (Figure 4, Table S3). The classes found to be dominant across all samples included Tremellomycetes (32.87%), Sordariomycetes (21.71%), Dothiomycetes (16.95%), Saccharomycetes (10.77%), and Agaricomycetes (1.05%) (Table S4). Although the proportion of dominant intestinal fungal phyla was different in different seasons, Ascomycota was found in higher abundance during fall and winter than spring (*p* = 0.002). Basidiomycota was found in higher abundance during spring (*p* < 0.001). The relative abundance of Zygomycota and Rozellomycota was higher in winter than in the other two seasons (*p* < 0.001; Figure 4).

**Figure 5:**
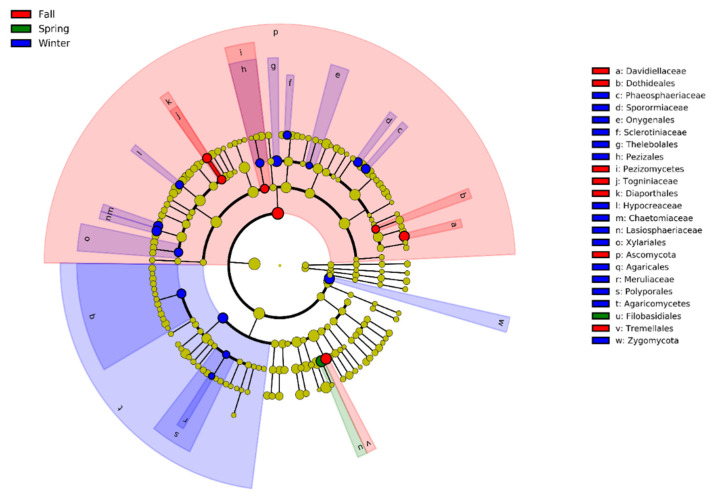
Linear discriminant analysis (LDA) effect size (LEfSe) analysis of the gut fungal biomarker of the hooded crane during fall, winter, and spring. Cladogram exhibits the biomarkers with the hierarchical taxonomic structure identified during three seasons. Evaluated biomarkers were ranked by effect size (LDA > 2, *p* < 0.05). Red phylotypes represent in fall; green phylotypes represent in spring; blue phylotypes represent in winter.

LEfSe was performed to determine the specific intestinal fungal taxa that varied across the three seasons. Six orders (Onygenales, Thelebolales, Pezizales, Xylariales, Agaricales, and Polyporales) and seven families (Phaeosphaeriaceae, Sporormiaceae, Sclerotiniaceae, Hypocreaceae, Chaetomiaceae, Lasiosphaeriaceae, and Meruliaceae) were significantly more abundant in winter. Only one order (Filobasidiales) was abundant in spring. Three orders (Dothediales, Diapothales, and Tremellales) and two families (Davidiellaceae and Togniniaceae) were more abundant in the fall (Figure 5, Figure S1).

**Figure 6:**
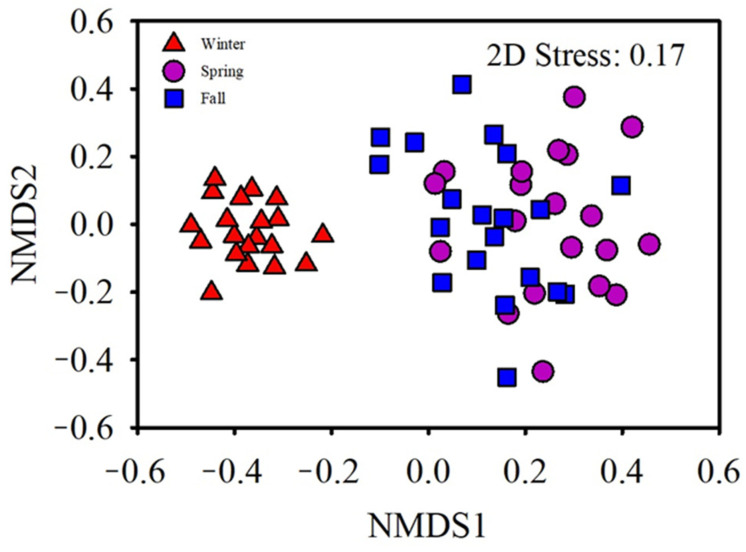
Gut fungal community structure of the hooded crane by applying non- metric multidimensional scaling (NMDS) amid fall, winter, and spring.

The result of the SIMPER analysis indicated that OTU_2396 (Nectriaceae; 6.93%), OTU_24 (*Cystofilobasidium*; 7.3%), OTU_2713 (Filobasidiales; 10.99%), and OTU_442 (*Guehomyces pullulans*; 5.14%) had a critical effect on the community difference between the spring and fall. OTU_2396 (Nectriaceae; 5%), OTU_24 (*Cystofilobasidium*; 7.42%), OTU_1970 (*Candida anthensensis*; 7.2%) and OTU_ 3281 (*Alternaria*; 4.71%) were involved in community difference between the winter and spring. The OTU_2396 (Nectriaceae; 1.1%), OTU_2173 (Filobasidiales; 1.21%), OTU_1970 (*Candida anthensensis*; 1.05%), OTU_442 (*Guehomyces pullulans*; 5.98%) and OTU_2729 (*Guehomyces pullulans*; 7.47%) were responsible for the community difference between the fall and winter (Table 1). To observe the seasonal effect on the gut fungal community of the hooded crane, ANOSIM analysis and NMDS were performed (*p* = 0.001; Table 2). The results indicated that seasonality affects the fungal community composition in hooded cranes. There were significant differences observed in the gut fungal community composition among the fall, winter, and spring seasons (Figure 6). The fungal OTUs associated with the three seasons were identified by indicator analysis. Overall, 28 indicator species were identified in winter, 11 in spring, and 22 in fall (Table S5).

### 3.3. Gut Fungal Pathogens

**Figure 7:**
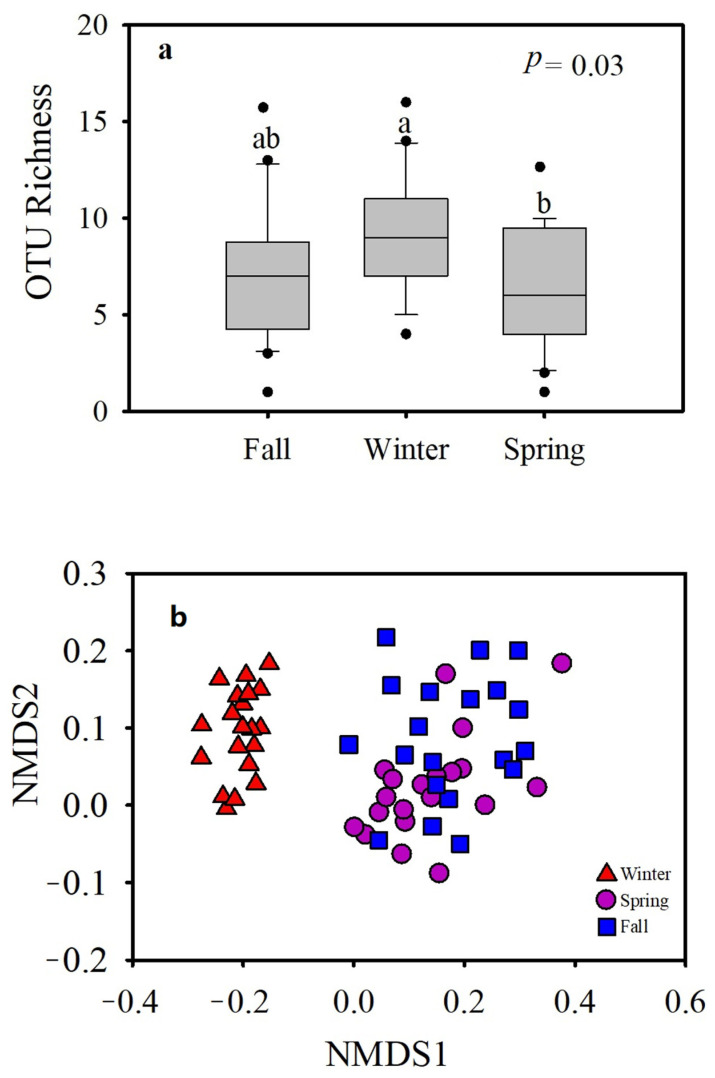
The potential gut fungal pathogenic OTU richness and community composition of the hooded crane during fall, winter, and spring. (**a**) Operational taxonomic unit (OTU) richness, and (**b**) analysis of similarity (ANOSIM) and Kruskal–Wallis Test. Different letters “a” and “b” represent significant differences (*p* < 0.05).

**Figure 8:**
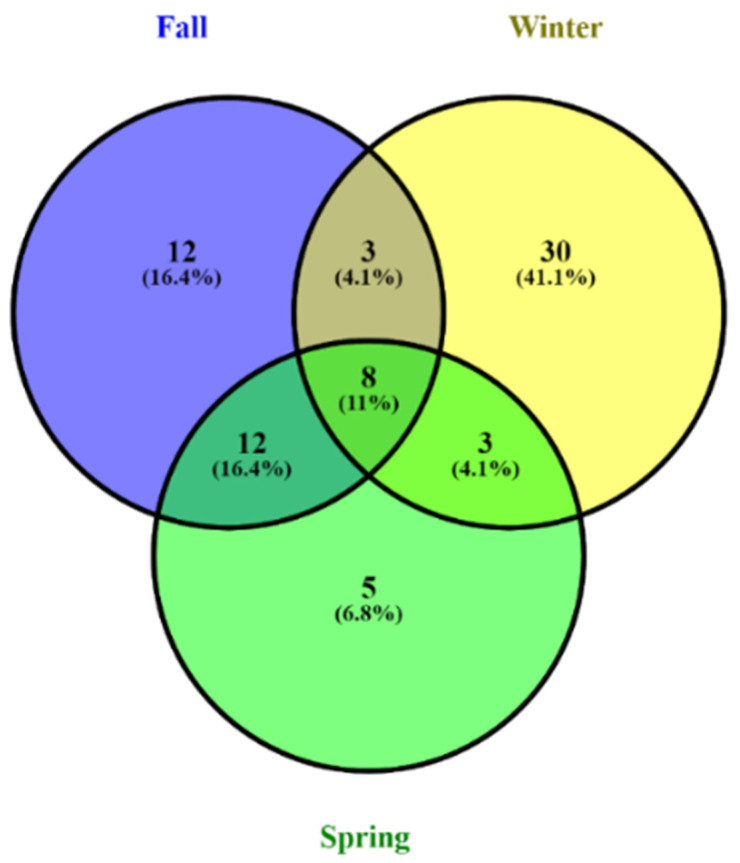
Analysis of the Venn diagram showing the unique and shared gut pathogenic fungal operational taxonomic units (OTUs) of the hooded crane during fall, winter, and spring.

A total of 454 (0.01 % relative to all fungal reads) potentially pathogenic reads were found across all samples of the three seasons, ranging from 0 to 16 sequences per sample (Table S7). The OTU richness for fungal pathogens was higher in winter samples than in spring and fall samples (Figure 7). The analysis of similarity indicated that the pathogenic fungal community was more significantly diverse in the winter season than the spring and fall seasons (ANOSIM *p =* 0.001) (Table 3, Figure 7). A total of 73 pathogenic OTUs were found; 8 (11%) were found in all seasons. During winter and spring, the shared fungal OTUs were 3 (4.1%); in fall and winter, the shared fungal OTUs were 3 (4.1%); during spring and fall, the shared fungal OTUs were 12 (16.4%). The unique OTUs found in fall, winter, and spring were 12 (16.4%), 30 (41.1%), and 5 (6.8 %), respectively (Figure 8).

The relative abundance of *Rhodotorula mucilaginosa*, *Aspergillus flavus, Aspergillus niger*, *Mortierella* sp, *Fusarium tricinctum,* and *Giberella fujikuroi* was high in winter, spring, and fall. *Phoma paspali* was high in abundance in fall relative to winter and spring. *Pencillium oxalicum* was high in abundance in winter and spring compared with fall. *Cryptococcus albidus* was relatively high in fall and spring compared with winter. The dominant pathogenic species found in the gut of the hooded crane across the three seasons might cause disease in humans, birds, animals, and plants (Table S6).

## 4. Discussion

Migratory birds exhibit a particular annual life cycle, including migration at various seasons with intricate dietary habits, exposure to the variable environment, and habitat type [10]. Birds have high-energy needs for their normal physiological functions; therefore, they may rely on their gut microbiota during seasonal migration. The variable food resources and habitats during seasonal migration play a crucial role and influence the gut microbiota of migratory birds [28]. The guts of birds harbor fungal taxa along with bacteria. In this study, the dominant fungal phyla found were Ascomycota, Basidiomycota, Zygomycota, and Rozellomycota across the winter, fall, and spring seasons. The results indicate that the gut fungal diversity and community composition showed more significant shifts during the winter season at the wintering ground than at the stopover sites during the fall and spring seasons. We also investigated the difference in gut fungal community composition during fall and spring at the stopover site. The study showed that the risk of cross-transmission of potential fungal pathogens might increase at wintering site because they are a shared environment with other wild birds and poultry.

The gut microbiota contributes to retaining the digestive function of wild birds during seasonal migration [29]. Ascomycota is the large phylum of the fungi kingdom, as it has been reported in various hosts, predominantly in dogs [30], cats [31], mice [32], fishes [33], insects [34], humans [35], and geese [36]. It plays an essential role in the physiology, growth, and metabolism mechanisms of host species and produces some crucial enzymes that aid in the digestion of complex carbohydrates [37]. The dominant classes of phylum Ascomycota found in the gut of the hooded crane across winter, fall, and spring were Sordariomycetes (21.6%), Dothideomycetes (16.6%), and Saccharomycetes (10.7%). The dominant phylum Basidiomycota, followed by dominant class Tremellomycetes (32.8%), were also detected in the gut of the hooded crane across the three seasons. These anaerobic fungi are competent in producing hydrolytic enzymes to degrade plant material, and thus, to increase the host’s digestion efficiency [38].

The gut fungal diversity and community composition exhibited significant shifts between the wintering and stopover grounds. During the extended winter season, Shengjin Lake provides the hooded crane with food resources, i.e., *Vallisneria natans, Potamogeton malainus*, *Phalaris arundinacea*, *Cynodom dactylon*, *Carex unisexualis*, *Cyperus* sp., *Polygonum lapathifolium*, rice grains, wheat seedlings, and invertebrates such as mollusks [15]. Some of the foraging habitats for wild birds have been degraded in the past because of the disturbance caused by humans [39]. It has been demonstrated that the gut microbiota undergoes significant changes when there come variations in bird food [40]. The alteration in foraging habitats allows the hooded crane to change their dietary structure during the extended winter period at Shengjin Lake [41]. Research has shown that wintering hooded cranes possess some saprophytic fungi in their gut, increasing the hooded cranes’ digestive efficiency to retain nutrient and energy balance during the extended winter period [6]. Therefore, the results indicating a significant difference in the gut fungal diversity and community composition of hooded cranes between the wintering and stopover sites corresponds with previous research. Studies have elucidated that corn (*Zea mays*) is a major food available at Lindian (stopover site) [17,42]. *Zea mays* is the only crop grown at Lindian every year. During fall, the corn crop is ready to harvest. The availability of food might be relatively higher in fall compared with spring. Thus, before arriving at the stopover sites (Lindian) in fall from the breeding grounds, and in spring from the wintering grounds, the hooded cranes may have been subjected to various dietary sources and environments [10]. Therefore, it is speculated that the gut fungal community composition of the hooded crane shows a significant difference during fall and spring due to the variable food resources and habitats of the cranes before arriving at the stopover sites.

In this study, a total of 73 fungal pathogenic OTUs were found in the hooded crane samples across the three seasons. The diversity and composition of the fungal pathogenic communities were significantly different in the winter relative to the fall and spring (Figure 6, Table 3). Migratory birds have exposure to environmental microbes during the migratory period and carry potential pathogens with them. Shengjin Lake, as an important wintering habitat for waterbirds, provides the birds with variable habitat types and food resources, and many migratory birds arrive during the winter period [13]. However, there is a niche overlap between the migratory birds and domestic birds, responsible for the cross-transmission of fungal pathogens [16]. Studies have illustrated that migratory birds are suspected of pathogenic invasion during seasonal migration [6]. During the long winter period, the hooded crane spends more time-sharing food resources with domestic poultry and other wild birds [16]. The risk of cross-transmission of potential fungal pathogens between migratory birds, domestic poultry, and other wild birds might be higher during the extended winter period.

The fungal pathogens higher in relative abundance in the winter were *Aspergillus niger* and *Aspergillus flavus* (Table 4). *Aspergillus niger* causes aspergillosis in humans, animals (dogs, cats, marine mammals), poultry, and other birds [43]. *Aspergillus flavus* is mainly responsible for causing aspergillosis, fungal endocarditis, pericarditis, urinary tract infection, CNS infections, rhinosinusitis and osteoarticular infection in humans [44], aspergillosis and respiratory tract infections in poultry [45], kernel rot in maize plants [46] yellow spot disease in tomato plants, yellow mold, ear rot in peanuts, and boll rot in cotton [47,48] (Table S6). *Phoma paspali* was higher in relative abundance in the gut of the hooded crane during the fall season than the winter and spring seasons (Table 4). It has been elucidated in studies that *Phoma paspali* was identified as a plant pathogen causing twisted leaf disease in sugarcane [49], and as responsible for subcutaneous abscess in humans [50]. *Cryptococcus albidus* was relatively high in abundance in fall and spring compared with winter in this study (Table 4). Studies reported that *Cryptococcus albidus* is a causative agent of fungemia and infection of the central nervous system [51], of cryptococcosis in birds such as pigeons, and of feline systemic diseases in cats [52,53] (Table S6). Some potential pathogenic species found in this study are also responsible for infections in humans. As migratory birds are known as carriers of potential pathogens, they might propagate the gut fungal pathogens to humans directly or through indirect sources during seasonal migration. The gut pathogens are potentially transmitted directly to humans who handle wild birds [54] or through contaminated air, water, or soil with feces of wild birds [16].

There were some limitations present in this work. Only one species with 60 samples was analyzed in this study. The samples of food resources were not collected and analyzed to clarify that diet resource differences at the wintering and stopover sites are what affect the gut fungal community composition of hooded cranes. The analysis and evaluations for certain limitations should be extended in future studies.

## 5. Conclusions

The intestinal fungal diversity significantly shifts during the winter season at wintering sites compared with the spring and fall seasons at the stopover site. The exposure with variable dietary sources, the habitat type, and the shared environment with other wild birds and domestic poultry during the prolonged harsh and dry winter period might be the potential causes for the significant shifts in gut fungal diversity between the wintering sites and stopover sites during the fall and spring. Differences and/or abundance in food resources might be responsible for the divergence in the gut fungal communities during winter, fall, and spring. The hooded crane might be at high risk of cross-transmission of fungal pathogens at the wintering sites. They share the same environment and food resources with other wild birds and domestic poultry during the extended winter period. Our work provides a complete sketch about seasonal variation in the gut fungal microbiome of hooded cranes at wintering and stopover sites during seasonal migration. Moreover, it also suggests that conservational strategies should be applied to prevent the transmission of potential pathogens between migratory birds, poultry, and other wild birds.

## Figures and Tables

**Table 1:** Similarity percentage analysis denotes the specific gut fungal OTUs of the hooded crane, determining the difference during the three seasons. Taxonomic abbreviations: f, family, o, order, g, genus, s, species.

OTU	Taxa	Contribution (%)		
		Spring vs. Fall	Spring vs. Winter	Fall vs. Winter
2396	f__ Nectriaceae	6.93	5	1.1
24	g__ *Cystofilobasidum*	7.3	7.42	-
2173	o__ Filobasidiales	10.99	-	1.21
1970	s__ *Candida anthensensis*	-	7.2	1.05
442	s__*Guehomyces pullulans*	5.14	-	5.98
3281	g__ *Alternaria*	-	4.71	-
2729	s__*Guehomyces pullulans*	-	-	7.47

**Table 2:** Analysis of similarity (ANOSIM) indicates the difference in the gut fungal community composition of the hooded crane samples amid the three seasons.

Treatment	ANOSIM	
	*r*	*p*
Fall vs. Winter	0.9285	0.001
Fall vs. Spring	0.2735	0.001
Winter vs. Spring	0.9201	0.001

**Table 3:** ANOSIM indicates the difference in gut fungal pathogenic community composition of the hooded crane amid the three seasons.

Treatment	ANOSIM	
	*r*	*p*
Fall vs. Winter	0.559	0.001
Fall vs. Spring	0.076	0.012
Winter vs. Spring	0.624	0.001

**Table 4:** The difference in the relative abundance of gut fungal pathogenic species of the hooded crane across the fall, winter, and spring seasons.

Pathogenic Species	Fall	Winter	Spring
*Rhodotorula mucilaginosa*	0.000(0.000) ^b^	0.3(0.470) ^a^	0.000(0.000) ^b^
*Aspergillus flavus*	0.000(0.000) ^b^	1.3(2.848) ^a^	0.000(0.000) ^b^
*Aspergillus niger*	0.000(0.000) ^b^	3.85(5.060) ^a^	0.000(0.000) ^b^
*Mortierella* sp.	0.000(0.000) ^b^	0.04(0.502) ^a^	0.05(0.223) ^b^
*Fusarium tricinctum*	0.000(0.000) ^b^	0.2(0.410) ^a^	0.05(0.223) ^b^
*Giberella fujikuro*	0.35(0.489) ^b^	0.8(0.523) ^a^	0.55(0.510) ^b^
*Pencillium oxalicum*	0.35(0.489) ^b^	0.8(0.523) ^a^	0.55(0.510) ^a b^
*Cryptococcus albidus*	2.10(1.774) ^a^	0.2(0.410) ^b^	2.00(1.450) ^a^
*Phoma paspali*	1.65(1.089) ^a^	0.7(0.671) ^b^	0.000(0.000) ^c^
*Candida tropicals*	0.95(2.928) ^a^	0.4(1.569) ^a^	0.55(1.571) ^a^
*Candida albicans*	153.65(479.61) ^a^	0.1(0.307) ^a^	0.3(0.97) ^a^
*Alternaria* sp.	0.9(0.718) ^a^	1.1(0.307) ^a^	1.00(0.000) ^a^
*Fusarium* sp.	0.15(0.670) ^a^	0.000(0.000) ^a^	0.000(0.000) ^a^
*Guehomyces pullulans*	1.65(1.089) ^a^	1.6(1.046) ^a^	1.00(0.858) ^a^
*Gibberella intricans*	0.8(0.833) ^a^	0.9(0.640) ^a^	0.7(0.732) ^a^
*Aspergillus subversicolor*	0.15(0.366) ^a^	0.000(0.000) ^a^	0.000(0.000) ^a^
*Aspergillus terreus*	0.000(0.000) ^a^	0.05(0.223) ^a^	0.1(0.307) ^a^
*Aspergillus fumigatus*	0.000(0.000) ^a^	0.1(0.307) ^a^	0.05(0.223) ^a^
*Aspergillus caesiellus*	0.05(0.223) ^a^	0.1(0.307) ^a^	0.05(0.223) ^a^
*Mortierella parvispora*	0.000(0.000) ^a^	0.1(0.307) ^a^	0.000(0.000) ^a^
*Gibberella zeae*	0.1(0.307) ^a^	0.000(0.000) ^a^	0.05(0.223) ^a^
*Fusarium kyushuense*	0.000(0.000) ^a^	4.5(20.124) ^a^	0.000(0.000) ^a^
*Fusarium pseudensiforme*	0.05(0.223) ^a^	0.05(0.223) ^a^	0.000(0.000) ^a^
*Fusarium solani* sp.	0.000(0.000) ^a^	0.1(0.307) ^a^	0.000(0.000) ^a^
*Aspergillus clavatus*	0.8(1.765) ^a^	1.65(4.760) ^a^	1.95(2.874) ^a^
*Mortierella parvispora*	0.000(0.000) ^a^	0.1(0.307) ^a^	0.000(0.000) ^a^

Standard deviation is denoted by values in brackets. Different letters “a”, “b” following the brackets represent significant differences from the Kruskal–Wallis test (*p* < 0.05).

## Data Availability

The raw data has been submitted to NCBI Sequence Read Archive (accession no PRJNA697866).

## Supplementary Materials

The following are available online at https://www.mdpi.com/article/10.3390/ani11040941/s1, Table S1: Analysis of data distribution of the hooded crane samples by applying the Kolmogorov–Smirnov test, Table S2: Sequencing and classification data of gut fungal OTUs of the hooded crane across winter, spring, and fall, Table S3: Dominant gut fungal phyla of the hooded crane with relative abundance across the three seasons in all samples, Table S4: Dominant gut fungal class of the hooded crane with relative abundance across the three seasons in all samples, Table S5: Indicator gut fungal OTUs of the hooded crane across fall, winter, and spring. Taxonomic abbreviation: f, family, o, order, g, genus, s, species, Table S6: Gut fungal pathogens identified in the gut of the hooded crane with target species and symptoms, Table S7: The gut fungal and potentially pathogenic sequences in the hooded crane samples during fall, winter, and spring, Figure S1: LDA score of the hooded crane samples during fall, winter, and spring. Identified biomarkers ranked by effect size (LDA > 2, *p* < 0.05).

## References

[1] Cani P.D. (2018). Human gut microbiome: Hopes, threats, and promises. Gut.

[2] Durack J., Lynch S.V. (2019). The gut microbiome: Relationships with disease and opportunities for therapy. J. Exp. Med..

[3] Ianiro G., Bruno G., Lopetuso L., Bartoli Beghella F., Laterza L., D’Aversa F., Gigante G., Cammarota G., Gasbarrini A. (2014). Role of yeasts in healthy and impaired gut microbiota: The gut mycome. Curr. Pharm. Des..

[4] Paterson M.J., Oh S., Underhill D.M. (2017). Host–microbe interactions: Commensal fungi in the gut. Curr. Opin. Microbiol..

[5] Chen M., Chen X.Q., Tian L.X., Liu Y.J., Niu J. (2020). Enhanced intestinal health, immune responses and ammonia resistance in Pacific white shrimp (*Litopenaeus vannamei*) fed dietary hydrolyzed yeast (*Rhodotorula mucilaginosa*) and Bacillus licheniformis. Aquac.Rep..

[6] Xiang X., Jin L., Yang Z., Zhang N., Zhang F. (2021). Dramatic shifts in intestinal fungal community between wintering Hooded Crane and Domestic Goose. Avian Res..

[7] Kamada N., Chen G.Y., Inohara N., Nunez G. (2013). Control of pathogens and pathobionts by the gut microbiota. Nat. Immunol..

[8] Cui L., Morris A., Ghedin E. (2013). The human mycobiome in health and disease. Genome Med..

[9] Byrd J.A., Caldwell D.Y., Nisbet D.J. (2017). The identification of fungi collected from the ceca of commercial poultry. Poult. Sci..

[10] Zhang F., Xiang X., Dong Y., Yan S., Song Y., Zhou L. (2020). Significant differences in the gut bacterial communities of Hooded Crane (*Grus monacha*) in different seasons at a stopover site on the flyway. Animals.

[11] Hume M.E., Hernandez C.A., Barbosa N.A., Sakomura N.K., Dowd S.E., Oviedo-Rondón E.O. (2012). Molecular identification and characterization of ileal and cecal fungus communities in broilers given probiotics, specific essential oil blends, and under mixed Eimeria infection. Foodborne Pathog. Dis..

[12] BirdLife International *Grus monacha*. IUCN Red List of Threatened Species. http://www.iucnredlist.org.

[13] Zheng M., Zhou L., Zhao N., Xu W. (2015). Effects of variation in food resources on foraging habitat use by wintering Hooded Cranes (*Grus monacha*). Avian Res..

[14] Wan W., Zhou L., Song Y. (2016). Shifts in foraging behavior of wintering Hooded Cranes (*Grus monacha*) in three different habitats at Shengjin Lake, China. Avian Res..

[15] Zhao F., Zhou L., Xu W., Zhao F., Zhou L., Xu W. (2013). Habitat utilization and resource partitioning of wintering Hooded Cranes and three goose species at Shengjin Lake. Chin. Birds.

[16] Xiang X., Zhang F., Fu R., Yan S., Zhou L. (2019). Significant differences in bacterial and potentially pathogenic communities between sympatric hooded crane and greater white-fronted goose. Front. Microbiol..

[17] Yang T., Huettmann F., Guo Y. (2014). Using stochastic gradient boosting to infer stopover habitat election and distribution of Hooded Cranes (*Grus monacha*) during spring migration in Lindian, Northeast China. PLoS ONE.

[18] Fang J., Wang Z., Zhao S., Li Y., Tang Z., Yu D., Ni L., Liu H., Xie P., Da L. (2006). Biodiversity changes in the lakes of the Central Yangtze. Front. Ecol. Environ..

[19] Cao L., Barter M.A., Lei G. (2008). New population estimates for Anatidae spending the non-breeding season in eastern China: Implications for flyway population estimates. Biol. Conserv..

[20] Yao H., Song J., Liu C., Luo K., Han J., Li Y., Pang X., Xu H., Zhu Y., Chen S. (2010). Use of ITS2 region as the universal DNA barcode for plants and animals. PLoS ONE.

[21] De Beeck M.O., Lievens B., Busschaert P., Declerck S., Vangronsveld J., Colpaert J.V. (2014). Comparison and validation of some ITS primer pairs useful for fungal metabarcoding studies. PLoS ONE.

[22] Caporaso J.G., Kuczynski J., Stombaugh J., Bittinger K., Bushman F.D., Costello E.K., Fierer N., Pena A.G., Goodrich J.K., Gordon J.I. (2010). QIIME allows analysis of high-throughput community sequencing data. Nat. Methods.

[23] Amir A., McDonald D., Navas-Molina J.A., Kopylova E., Morton J.T., Xu Z.Z., Kightley E.P., Thompson L.R., Hyde E.R., Gonzalez A. (2017). Deblur rapidly resolves single-nucleotide community sequence patterns. mSystems.

[24] Edgar R.C. (2010). Search and clustering orders of magnitude faster than BLAST. Bioinformatics.

[25] Segata N., Izard J., Waldron L., Gevers D., Miropolsky L., Garrett W.S., Huttenhower C. (2011). Metagenomic biomarker discovery and explanation. Genome Biol..

[26] Anderson M.J., Walsh D.C. (2013). PERMANOVA, ANOSIM, and the Mantel test in the face of heterogeneous dispersions: What null hypothesis are you testing?. Ecol. Monogr..

[27] Gibert C., Escarguel G. (2019). PER-SIMPER—A new tool for inferring community assembly processes from taxon occurrences. Glob. Ecol. Biogeogr..

[28] Lewis W.B., Moore F.R., Wang S. (2016). Characterization of the gut microbiota of migratory passerines during stopover along the northern coast of the Gulf of Mexico. J. Avian Biol..

[29] McWilliams S.R., Karasov W.H. (2001). Phenotypic flexibility in digestive system structure and function in migratory birds and its ecological significance. Comp. Biochem. Physiol. A Mol. Integr. Physiol..

[30] Foster M.L., Dowd S.E., Stephenson C., Steiner J.M., Suchodolski J.S. (2013). Characterization of the fungal microbiome (mycobiome) in fecal samples from dogs. Vet. Med. Int..

[31] Meason-Smith C., Diesel A., Patterson A.P., Older C.E., Johnson T.J., Mansell J.M., Suchodolskit J.S., Hoffmann A.R. (2017). Characterization of the cutaneous mycobiota in healthy and allergic cats using next generation sequencing. Adv. Vet. Dermatol..

[32] Qiu X., Zhang F., Yang X., Wu N., Jiang W., Li X., Li X., Liu Y. (2015). Changes in the composition of intestinal fungi and their role in mice with dextran sulfate sodium-induced colitis. Sci. Rep..

[33] Gatesoupe F.J. (2007). Live yeasts in the gut: Natural occurrence, dietary introduction, and their effects on fish health and development. Aquaculture.

[34] Shao M.W., Lu Y.H., Miao S., Zhang Y., Chen T.T., Zhang Y.L. (2015). Diversity, bacterial symbionts and antibacterial potential of gut-associated fungi isolated from the *Pantala flavescens* larvae in China. PLoS ONE.

[35] Huffnagle G.B., Noverr M.C. (2013). The emerging world of the fungal microbiome. Trends Microbiol..

[36] Wu Y., Yang Y., Cao L., Yin H., Xu M., Wang Z., Liu Y., Wang X., Deng Y. (2018). Habitat environments impacted the gut microbiome of long-distance migratory swan geese but central species conserved. Sci. Rep..

[37] Siriyappagouder P., Kiron V., Lokesh J., Rajeish M., Kopp M., Fernandes J. (2018). The intestinal mycobiota in wild zebrafish comprises mainly Dothideomycetes while Saccharomycetes predominate in their laboratory-reared counterparts. Front. Microbiol..

[38] Ljungdahl L.G. (2008). The cellulase/hemicellulase system of the anaerobic fungus Orpinomyces PC-2 and aspects of its applied use. Ann. N. Y. Acad. Sci..

[39] Zhao G.H., Zhou L.Z., Dong Y.Q., Cheng Y.Y., Song Y.W. (2017). The gut microbiome of hooded cranes (*Grus monacha*) wintering at Shengiin Lake, China. MicrobiologyOpen.

[40] Semova I., Carten J.D., Stombaugh J., Mackey L.C., Knight R., Farber S.A. (2012). Microbiota regulate intestinal absorption and metabolism of fatty acids in the zebrafish. Cell Host Microbe.

[41] Zhang N., Zhou L., Yang Z., Gu J. (2021). Effects of Food changes on intestinal bacterial diversity of wintering hooded cranes (*Grus monacha*). Animals.

[42] Jian H., Yumin G. (2015). Diet of hooded crane (*Grus monacha*) in Autumn, Lindian, China. Chin. J. Wildl..

[43] Seyedmousavi S., Guillot J., Arné P., De Hoog G.S., Mouton J.W., Melchers W.J., Verweij P.E. (2015). Aspergillus and aspergilloses in wild and domestic animals: A global health concern with parallels to human disease. Med. Mycol..

[44] Hedayati M.T., Pasqualotto A.C., Warn P.A., Bowyer P., Denning D.W. (2007). *Aspergillus flavus*: Human pathogen, allergen and mycotoxin producer. Microbiology.

[45] Dorner J.W., Cole R.J., Lomax L.G., Gosser H.S., Diener U.L. (1983). Cyclopiazonic acid production by *Aspergillus flavus* and its effects on broiler chickens. Appl. Environ. Microbiol..

[46] Dolezal A.L., Shu X., OBrian G.R., Nielsen D.M., Woloshuk C.P., Boston R.S., Payne G.A. (2014). *Aspergillus flavus* infection induces transcriptional and physical changes in developing maize kernels. Front. Microbiol..

[47] Klich M.A. (2007). *Aspergillus flavus*: The major producer of aflatoxin. Mol. Plant Pathol..

[48] Abrar A., Ali Z., Mughal T.A., Malik K., Sarwar S., Oneeb M., Abass M., Qamar H., Nasir R. (2020). Effects of entomopathogenic *Aspergillus flavus* on tomato plant (*Solanum lycopersicum*) endophytic activity under agro-climatic condition of Lahore, Punjab-Pakistan. Pure Appl. Biol..

[49] Lin Z., Wei J., Zhang M., Xu S., Guo Q., Wang X., Wang J., Chen B., Que Y., Deng Z. (2015). Identification and characterization of a new fungal pathogen causing twisted leaf disease of sugarcane in China. Plant Dis..

[50] Young N.A., Kwon-Chung K.J., Freeman J. (1973). Subcutaneous abscess caused by *Plioma* sp. resembling Pyrenochacla romeroi: Unique fungal infection occurring in immunosuppressed recipient of renal allograft. Am. J. Clin. Pathol..

[51] Choe Y.J., Blatt D.B., Yalcindag A., Geffert S.F., Bobenchik A.M., Michelow I.C. (2020). *Cryptococcus albidus* fungemia in an immunosuppressed child: Case report and systematic literature review. Pediatr. Infect. Dis. J..

[52] Werther K., de Sousa E., Júnior J.R.A., Ardisson F.A., Giannini M.J. (2011). *Cryptococcus gattii* and *Cryptococcus albidus* in captive domestic pigeon (*Columba livia*). Depression.

[53] Kano R., Kitagawa M., Oota S., Oosumi T., Murakami Y., Tokuriki M., Hasegawa A. (2008). First case of feline systemic *Cryptococcus albidus* infection. Med. Mycol..

[54] Abulreesh H.H., Goulder R., Scott G.W. (2007). Wild birds and human pathogens in the context of ringing and migration. Ring. Migr..

