# Supplementary material for: Seasonal Variations in the Gut Fungal Communities of Hooded Crane (Grus monacha) at Wintering and Stopover Sites in China
Source: Animals (Basel). 2021 Mar 26;11(4):941. doi: 10.3390/ani11040941 (PMC8067105; doi:10.3390/ani11040941)
Supplement: Supplementary file 1 [file animals-11-00941-s001.pdf]

Table S1. Analysis of data distribution of hooded crane samples by applying the Kolmogorov-Smirnov test.

	**Kolmogorov-Smirnov test (*P* value)**	**Distribution**
Fungal OTU richness	0.492	Normal
Fungal Chao 1	0.287	Normal
Fungal Shannon	0.533	Normal
Fungal simpson	0.000	Non-normal
Ascomycota	0.640	Normal
Basidiomycota	0.081	Normal
Rozellomycota	0.000	Non-normal
Zygomycota	0.824	Normal

Normal distribution: $P > 0.05$; Non-normal distribution: $P < 0.05$

Table S2. Sequencing and classification data of gut fungal OTUs of hooded crane across winter, spring, and fall.

Samples	Sequences	OTU	Phylum	Class	Order	Family	Genus
Winter1	49876	399	6	18	47	88	131
Winter2	47185	391	7	18	45	89	133
Winter3	47504	447	7	21	50	96	142
Winter4	56032	222	7	18	33	60	89
Winter5	54090	473	7	20	48	89	139
Winter6	54862	386	7	19	42	73	121
Winter7	61273	302	6	14	32	66	97
Winter8	54788	574	6	17	45	89	143
Winter9	72570	459	7	21	50	92	140
Winter10	63525	365	7	20	42	71	111
Winter11	62071	516	6	20	47	82	140
Winter12	68751	452	7	20	42	82	129
Winter13	58233	443	6	18	46	85	134
Winter14	61715	598	7	21	49	105	179
Winter15	70305	590	6	20	50	93	160
Winter16	48343	338	7	18	43	80	123
Winter17	67865	438	7	18	45	87	137
Winter18	70807	371	5	14	38	76	120
Winter19	59370	436	6	19	45	85	125
Winter20	72559	479	6	20	50	91	150
Spring1	58115	209	6	17	38	60	92
Spring2	44552	151	4	12	24	34	58
Spring3	68588	182	5	17	36	57	82
Spring4	70513	170	4	15	33	51	76
Spring5	59961	200	5	16	31	49	79
Spring6	65479	127	3	12	28	38	62
Spring7	63849	151	4	15	30	43	69
Spring8	71984	167	3	11	20	29	53
Spring9	62019	228	5	15	32	52	94
Spring10	54769	262	5	17	38	60	94
Spring11	70733	111	3	11	23	31	50
Spring12	72901	144	4	12	23	34	55

Spring13	70127	153	4	15	29	44	60
Spring14	64610	212	4	15	33	51	81
Spring15	29236	233	7	18	33	52	82
Spring16	66756	160	4	13	29	42	67
Spring17	67169	57	3	10	15	21	26
Spring18	74354	120	4	13	25	40	62
Spring19	71690	152	4	15	33	49	72
Spring20	73575	174	4	13	30	45	74
Fall1	59154	192	5	17	32	47	78
Fall2	73724	414	6	19	47	81	140
Fall3	66040	90	4	10	18	28	39
Fall4	66331	172	6	14	32	46	68
Fall5	60135	138	4	12	29	43	64
Fall6	67422	222	5	19	39	58	97
Fall7	59216	109	5	14	27	39	59
Fall8	58304	133	5	13	28	40	62
Fall9	37043	373	5	19	39	65	113
Fall10	39099	173	4	12	25	42	59
Fall11	53022	221	4	15	38	69	104
Fall12	63600	308	5	19	46	72	117
Fall13	32877	199	6	17	34	52	81
Fall14	70011	296	6	23	43	74	115
Fall15	71819	134	4	14	32	45	70
Fall16	30467	201	4	14	27	42	70
Fall17	73820	228	4	13	29	41	72
Fall18	37234	139	4	12	27	40	59
Fall19	63064	197	5	16	35	57	85
Fall20	58298	354	6	20	25	69	119

Table S3. Dominant gut fungal phyla of hooded crane with relative abundance across three seasons in all samples.

**Phylum**	**Distribution**	***P***	**Relative abundance (%)**		
			**Fall**	**Winter**	**Spring**
Ascomycota	Normal	0.002	72.58(28.11)^{a}	62.87 (13.80)^{a}	41.46 (33.05)^{b}
Basidiomycota	Normal	< 0.001	26.47 (28.64)^{b}	24.71 (12.98)^{b}	58.31 (33.13)^{a}
Rozellomycota	Non-Normal	< 0.001	0.00 (7.71)^{b}	3.17 (3.44)^{a}	0.00(0.02)^{b}
Zygomycota	Normal	< 0.001	0.70 (1.00)^{b}	6.6 (7.24)^{a}	0.18 (0.27)^{b}

The values in brackets represent the standard deviation of the mean. The distribution of data was analyzed by the Kolmogorov Smirnov test. Normal distribution: $P > 0.05$, One-way ANOVA. Different letters of ''a'' and b'' behind brackets represent significant differences from Tukey HSD comparisons ($P < 0.05$). Non-normal distribution: $P < 0.05$, KW. Different letters behind brackets represent significant differences from the Mann-Whitney-Wilcoxon test ($P < 0.05$).

Table S4. Dominant gut fungal class of hooded crane with relative abundance across three seasons in all samples.

**Class**	**Distribution**	***P***	**Relative abundance (%)**		
			**Fall**	**Winter**	**Spring**
Dothideomycetes	Normal	0.001	25.8 (0.24)^{a}	19.2 (0.09)^{a}	5 (0.08)^{b}
Saccharomycetes	Non-Normal	0.012	20.2(0.27)^{a}	0.0(0.000)^{b}	12.0(0.22)^{ab}
Sordariomycetes	Normal	0.53	19.1(0.19)^{a}	26.0(0.13)^{a}	19.8(0.28)^{a}
Agaricomycetes	Non-Normal	< 0.001	0.0(0.000)^{b}	3.0(0.10)^{a}	0.0(0.000)^{b}

Tremellomycetes	Normal	< 0.001	25.1(0.28)[b]	15.9(0.07)[b]	57.4 (0.33)[a]

The values in brackets represent the standard deviation of the mean. The distribution of data was analyzed by the Kolmogorov Smirnov test. Normal distribution: P > 0.05, One-way ANOVA. Different letters behind brackets represent significant differences from Tukey HSD comparisons (P < 0.05). Non-normal distribution: P < 0.05, KW. Different letters of ''a' and ''b'' behind brackets represent significant differences from the Mann-Whitney-Wilcoxon test (P < 0.05).

Table S5. Indicator gut fungal OTUs of hooded crane across fall, winter, and spring. Taxonomic abbreviation: f, family, o, order, g, genus, s, specie.

**Treatment**	**Indicator OTU**	**Relative abundance (%)**	***P* value**	**Taxonomy**
	OTU1970	8.000	0.007	s__*Candida athensensis*
	OTU3281	3.489	0.001	s__*Alternaria* sp.
	OTU2014	2.803	0.033	s__*Cryptococcus terrestris*
	OTU2773	1.806	0.001	s__*Davidiella tassiana*
	OTU2710	1.591	0.001	o__Pleosporales
	OTU2434	1.359	0.001	s__*Alternaria* sp.
	OTU706	0.354	0.002	s__*Phaeoacremonium hungaricum*
	OTU918	0.323	0.003	s__*Sporobolomyces oryzicola*
	OTU537	0.263	0.007	s__*Cryptococcus albidus*
	OTU3007	0.206	0.035	s__*Hannaella siamensis*
**Fall**	OTU3624	0.172	0.001	g__*Alternaria*
	OTU469	0.171	0.003	s__*Mucor circinelloides*
	OTU938	0.170	0.021	s__*Candida quercitrusa*
	OTU451	0.162	0.002	s__Davidiella tassiana
	OTU676	0.135	0.01	s__*Didymella exigua*
	OTU2753	0.100	0.019	c__Dothideomycetes
	OTU3624	0.172	0.001	g__*Alternaria*
	OTU1469	0.171	0.003	s__*Mucor circinelloides*
	OTU938	0.170	0.021	s__*Candida quercitrusa*
	OTU1451	0.162	0.002	s__*Davidiella tassiana*
	OTU1676	0.135	0.01	s__*Didymella exigua*
	OTU2753	0.100	0.019	c__Dothideomycetes
	OTU24	4.739	0.001	s__*Cystofilobasidium infirmominiatum*
	OTU2036	4.117	0.001	s__*Thelebolales* sp.
	OTU3239	3.017	0.001	s__*Phoma calidophila*
	OTU2707	2.817	0.001	s__*Acremonium nepalense*
	OTU2716	1.876	0.001	s__*Mortierella camargensis*
	OTU3528	1.796	0.001	s__*Acremonium dichromosporum*
	OTU933	1.463	0.001	s__*Acremonium dichromosporum*
	OTU2213	1.398	0.001	s__*Montagnulaceae* sp.
	OTU1865	0.947	0.001	s__*Rozellomycota* sp.
**Winter**	OTU2323	0.831	0.001	s__*Rhodotorula vanillica*
	OTU2205	0.703	0.006	s__*Ceratobasidiaceae* sp
	OTU2210	0.432	0.001	s__*Rhodotorula vanillica*
	OTU2338	0.408	0.001	s__*Phoma calidophila*
	OTU1547	0.337	0.001	f__Lasiosphaeriaceae
	OTU1441	0.206	0.035	s__*Sclerotium hydrophilum*
	OTU2246	0.195	0.001	s__*Leucosporidiella fragaria*
	OTU1169	0.192	0.001	s__*Mortierellales* sp.
	OTU1616	0.183	0.001	s__*Phoma calidophila*
	OTU2839	0.180	0.001	s__*Acremonium nepalense*
	OTU855	0.175	0.001	s__*Phoma calidophila*
	OTU2317	0.175	0.001	s__*Nomuraea rileyi*
	OTU3419	0.156	0.001	s__*Schizothecium carpinicola*
	OTU1934	0.126	0.001	s__*Mastigobasidium intermedium*
	OTU1570	0.121	0.001	s__*Stagonospora perfecta*
	OTU1937	0.115	0.001	s__*Mastigobasidium intermedium*
	OTU3610	0.108	0.001	s__*Acremonium dichromosporum*
	OTU3628	0.107	0.001	s__*Pseudeurotium hygrophilum*
	OTU1768	0.104	0.001	o__Pleosporales

Spring	OTU2713	8.785	0.001	s__*Filobasidiales* sp.
	OTU2729	5.143	0.012	s__*Guehomyces pullulans*
	OTU2937	2.486	0.001	s__*Cryptococcus victoriae*
	OTU1903	1.241	0.001	s__*Cystofilobasidium infirmominiatum*
	OTU2386	0.891	0.019	s__*Gibberella intricans*
	OTU1011	0.849	0.001	s__*Cryptococcus* sp SM13L02
	OTU1656	0.321	0.001	s__*Cryptococcus* sp SM13L02
	OTU1496	0.260	0.001	s__*Cryptococcus victoriae*
	OTU244	0.148	0.001	s__*Cryptococcus victoriae*
	OTU2241	0.119	0.004	s__*Rhodosporidium diobovatum*
	OTU2737	0.115	0.001	s__*Cryptococcus victoriae*

Table S6. Gut fungal pathogens identified in gut of hooded crane with target species and symptoms.

Pathogenic species	Infection	Target organism
Candida albicans	Candidiasis	Humans and Birds
Candida tropicalis	Candidiasis	Human and Birds
Cryptococcus albidus	Cryptococcosis, Feline systemic infection, Fungemia, CNS infections	Humans, Birds, Cats
Rhodotorula mucilaginosa	Fungemia, Epididymitis, etc	Humans, Sheep, Cattle
Alternaria sp.	Asthma, Pneumonitis, etc	Humans and Animals
Fusarium sp.	Keratomycosis, Mycotoxicosis	Humans and Animals
Fusarium kyushuense	Pneumonitis, Fusarium Wilt, etc	Humans, Plants, Mouse
Fusarium solani	Root rot	Plants
Fusarium tricinctum	Root rot	Plants
Fusarium pseudensiforme	Keratitis, Fusariosis	Humans
Aspergillus fumigatus	Aspergillosis, Mycotic pneumonia, Gastroenteritis	Birds, wild animals Mammals
Aspergillus flavus	Aspergillosis, endocarditis, pericarditis, urinary tract infection, CNS infections, Rhinosinusitis and Osteoarticular infections, Kernel rot, Yellow spot, Ear rot, Boll rot	Birds, Humans and plants
Aspergillus caesiellus	Aspergillosis, Pneumonia, Gastroenteritis	Birds, Wild animals, Mammals
Aspergillus clavatus	Aspergillosis	Birds, Wild animals, Mammals
Aspergillus niger	Aspergillosis	Birds, Wild animals, Mammals
Aspergillus terreus	Aspergillosis	Birds, Wild animals, Mammals
Aspergillus subversicolor	Aspergillosis	Birds, Wild animals, Mammals

Gibberella fujikuroi	Leukoencephalomalacia, Ear rot, Grain mold	Humans, Animals, Plants
Gibberella intricans	Keratitis, Scab Ear rot, etc	Humans, Animals, Plants
Gibberella zeae	Head blight, Mytotoxicosis	Humans, Animals, Plants
Penicillium critinum	Necrosis, Pneumonia	Birds, Humans and Animals
Penicillium oxalicum	Pneumonia, Keratitis, Blue mold	Humans, Animals, Plants
Guehomyces pullulans	Dermatitis, Abscess, Meningitis	Humans
Phoma papalis	Gangrene, Subcutaneous abscess, Keratitis, Crop root, Twisted leaf disease	Humans, Animals, Plants
Mortierella parvispora	Necrosis, Chlorosis	Soil, Plants

Table S7. The gut fungal and potentially pathogenic sequences in hooded crane samples during fall, winter, and spring.

**Samples**	**Sequence**		**Pathogen/Fungi (%)**
	**Fungi**	**Pathogen**	
Winter1	49876.0	14	0.028
Winter2	47185.0	10	0.029
Winter3	47504.0	8	0.016
Winter4	56032.0	4	0.024
Winter5	54090.0	5	0.025
Winter6	54862.0	10	0.025
Winter7	61273.0	5	0.022
Winter8	54788.0	9	0.025
Winter9	72570.0	9	0.019
Winter10	63525.0	7	0.022
Winter11	62071.0	13	0.022
Winter12	68751.0	5	0.020
Winter13	58233.0	11	0.024
Winter14	61715.0	16	0.022
Winter15	70305.0	13	0.019
Winter16	48343.0	9	0.028
Winter17	67865.0	5	0.020
Winter18	70807.0	5	0.019
Winter19	59370.0	11	0.023
Winter20	72559.0	9	0.019
Spring1	58115.0	6	0.024
Spring2	44552.0	2	0.031
Spring3	68588.0	8	0.020
Spring4	70513.0	7	0.019

Spring5	59961.0	10	0.023
Spring6	65479.0	5	0.021
Spring7	63849.0	5	0.021
Spring8	71984.0	7	0.019
Spring9	62019.0	10	0.022
Spring10	54769.0	10	0.025
Spring11	70733.0	6	0.019
Spring12	72901.0	10	0.019
Spring13	70127.0	6	0.019
Spring14	64610.0	7	0.021
Spring15	29236.0	10	0.047
Spring16	66756.0	4	0.020
Spring17	67169.0	3	0.020
Spring18	74354.0	4	0.018
Spring19	71690.0	6	0.019
Spring20	73575.0	3	0.019
Fall1	59154.0	8	0.023
Fall2	73724.0	16	0.018
Fall3	66040.0	4	0.021
Fall4	66331.0	3	0.021
Fall5	60135.0	4	0.023
Fall6	67422.0	7	0.020
Fall7	59216.0	1	0.023
Fall8	58304.0	7	0.024
Fall9	37043.08	8	0.037
Fall10	39099.0	0	0.035
Fall11	53022.0	7	0.026
Fall12	63600.0	5	0.022
Fall13	32877.0	5	0.042
Fall14	70011.0	11	0.019
Fall15	71819.0	4	0.019
Fall16	30467.0	11	0.045
Fall17	73820.0	7	0.018
Fall18	37234.0	7	0.037
Fall19	63064.0	6	0.022
Fall20	58298.0	13	0.024
**Total**	**3623384**	**454**	**0.012**

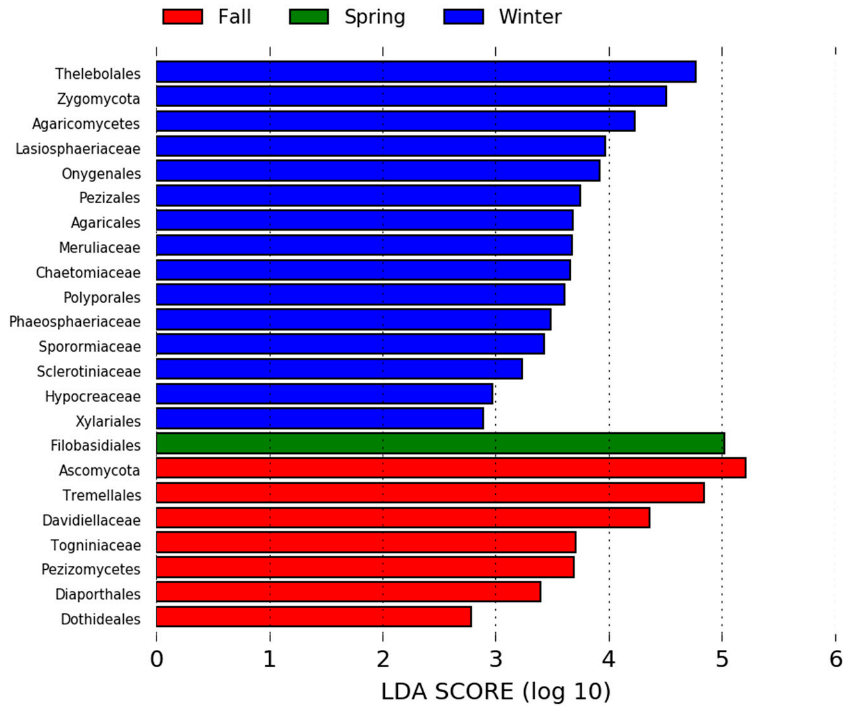

Figure S1. LDA score of hooded crane samples during fall, winter, and spring. Identified biomarkers ranked by effect size (LDA > 2, *P* < 0.05).
